# The Japan-UK Synthetic Biology Conference, Spring 2025: Strengthening Global Links to Engineer Biology

**DOI:** 10.1021/acssynbio.5c00232

**Published:** 2025-06-20

**Authors:** Thomas E. Gorochowski, Michael A. Brockhurst, Francesca Ceroni, Yuka W. Iwasaki, Nozomu Yachie

**Affiliations:** † School of Biological Sciences, 1980 University of Bristol, 24 Tyndall Avenue, Bristol BS8 1TQ, U.K.; ‡ Division of Evolution, Infection and Genomics, Faculty of Biology, Medicine and Health, 5292 University of Manchester, Manchester M13 9PL, U.K.; § Department of Chemical Engineering and Imperial College Centre for Synthetic Biology 4615 Imperial College London, London SW7 2AZ, U.K.; ∥ Imperial College Centre for Synthetic Biology, 4615 Imperial College London, London SW7 2AZ, U.K.; ⊥ Bezos Centre for Sustainable Proteins andNational Alternative Protein Innovation Centre (NAPIC), London SW7 2AZ, U.K.; # Laboratory for Functional Non-coding Genomics, 198286 RIKEN Center for Integrative Medical Sciences, 1-7-22 Suehiro-cho, Tsurumi-ku, Yokohama 230-0045, Japan; ⊗ School of Biomedical Engineering, Faculty of Applied Science and Faculty of Medicine, 8166 The University of British Columbia, Vancouver, British Columbia V6T 1Z3, Canada; ○ Premium Research Institute for Human Metaverse Medicine (WPI-PRIMe), Osaka University, Suita, Osaka 565-0871, Japan; ¶ Research Center for Advanced Science and Technology, The University of Tokyo, Tokyo 153-8904, Japan

**Keywords:** international collaboration, synthetic biology, engineering biology, UK, Japan

## Abstract

Both Japan and the UK have recognized the growing importance of synthetic and engineering biology for transforming life science research and transitioning toward a sustainable biobased economy. Such a shift will require extensive international cooperation and collaboration. In this viewpoint, we provide a summary of the recent “Japan-UK Synthetic Biology Conference, Spring 2025” that aimed to facilitate new links between researchers across the broad field of synthetic biology. We cover the core scientific topics discussed, distill some of the emerging trends, and outline the remaining challenges that are hampering progress. We end by highlighting some of the ways in which international collaborations may help address these issues through a combination of sharing expertise, national infrastructures, and aligned funding.

## Graphical abstract

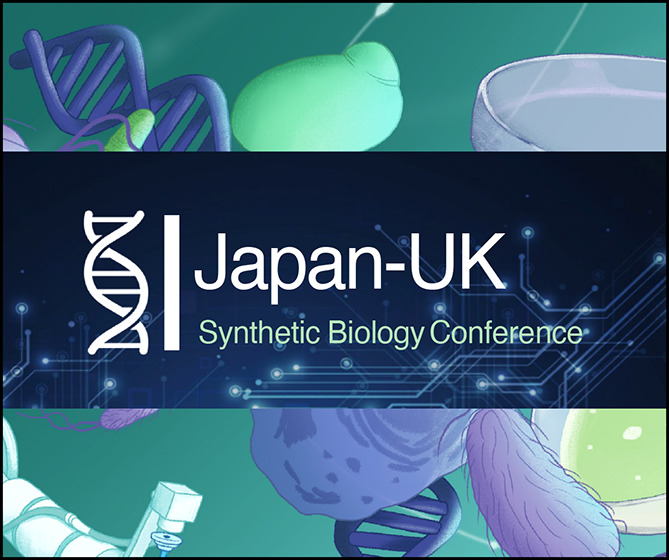

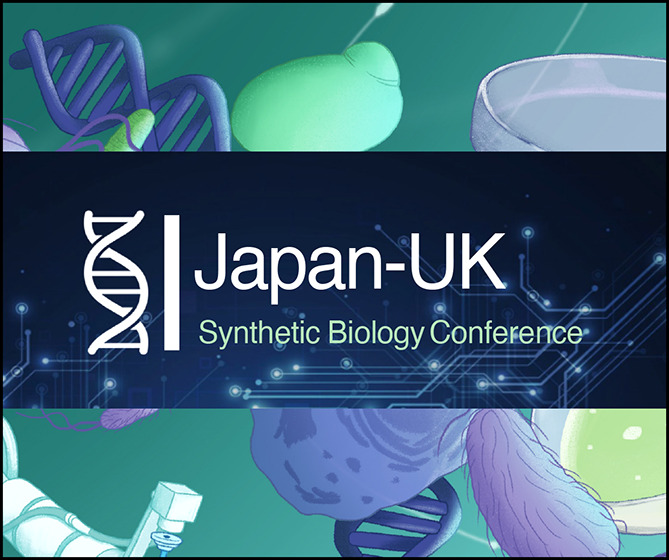

## Introduction

The “Japan-UK Synthetic Biology Conference, Spring 2025” took place in Osaka, Japan from the 3^rd^–4^th^ of February 2025, welcoming 22 participants from Japan and the UK with a broad spread of research interests spanning cell-free systems to synthetic genomics. Japanese representatives were mainly from synthetic biology-related funding programs of the Japan Science and Technology Agency (JST) (e.g., Genome Programming Program and Cell Control Program), but also included synthetic biologists from other leading Japanese institutions, such as The University of Tokyo and Osaka University. UK representatives included members of recent UK investments in the field, including the UKRI Engineering Biology Mission Hubs (e.g., Engineering Biology Hub for Microbial Foods, and Engineering Biology Hub for Environmental Processing and Recovery of Metals) and Mission Awards (e.g., CYBER: Cyanobacteria Engineering for Restoring Environments, and SafePhage: Engineering Synthetic Phages with Intrinsic Biocontainment), as well as established national centers for synthetic and engineering biology (e.g., SynbiCITE, and London DNA Foundry). Diversity across the event was seen not only in the science but also in the gender ratio and career stages of the participants, making for a vibrant and engaging event. The conference was structured around two full days of scientific talks and an additional trip for UK delegates to the newly established Institute for Advanced Study of Human Biology (ASHBi) at Kyoto University the day after the main conference. This visit included a tour of the facilities, discussions on the setup and structure of the institute, and a talk by Paul Freemont (Imperial College London, UK) on the evolution of synthetic biology within the UK research and innovation landscape.

In this viewpoint, we provide an overview of the topics covered throughout the conference, highlight the key points raised, and distill common themes from the discussions that suggest interesting directions for the field and possible future points for fruitful international collaboration between Japan and the UK.

## Cell-Free Systems

The scientific program started with a focus on bottom-up synthetic biology and the growing use of reconstituted cell-free systems for dissecting biological complexity and more precisely controlling engineered biological systems *in vitro*. The talks began with Paul Freemont (Imperial College London, UK) providing a broad introduction to the field, highlighting the numerous applications of cell-free systems to date.^1^ Freemont emphasized some of the major hurdles that remain when using cell-free systems, such as challenges in maintaining reactions for long periods of time (i.e., energy generation and recycling of resources) and batch-to-batch variability when using crude cell extracts. He then discussed some of the work his team has done to try and better understand optimal compositions of cell-free reactions^2^ and applications of synthetic biology and cell-free systems to accelerate research related to extracellular vesicles.^3^

Next, Yutetsu Kuruma (Japan Agency for Marine-Earth Science and Technology, Japan) asked the question, “What is a living system?” and, specifically, “Can we create a synthetic self-sustained cell?” He stressed how growth and division are key to answering these questions and described recent advances in his lab where the PURE cell-free expression system encapsulated in giant unilamellar vesicles (GUVs) was used to explore this further. He showed how combining fatty acid synthesis with the production of acyltransferases enabled the production of submillimolar concentrations of phospholipids in a single reaction.^4^ Interestingly, the synthesized phospholipids became localized to the membrane of the GUV, making this system an interesting platform for studying self-replicating synthetic cells where significant membrane expansion is required.

Norikazu Ichihashi (The University of Tokyo, Japan) built on these ideas, exploring how an *in vitro* central dogma could be used to reconstitute self-regenerating protein synthesis. Ichihashi’s focus was specifically on the need for charged tRNAs to enable this process. He discussed how all 20 aminoacyl-tRNA synthetases could be regenerated using the PURE system^5^ and how clever RNA processing methods can be used to allow for 21 tRNAs to be synthesized in a functional form from a single polycistronic DNA template.^6^ He also highlighted that while this research provides fundamental insights into protein translation, it also acts as a valuable platform for more significant modification of translational processes, especially those required for genetic code expansion.

The session ended with Yuta Kawashima and Kunio Okawa from the JST providing an overview of the synthetic biology funding landscape in Japan. They highlighted several mechanisms for future international collaborations between Japanese and UK researchers, such as through the Strategic International Collaborative Research Program (SICORP), Adopting Sustainable Partnerships for Innovative Research Ecosystem (ASPIRE) program and aligned JST/EMBO grants and fellowships. They also spoke about previously successful initiatives such as the recent joint JST/BBSRC “Japan-UK engineering biology for discovery research and cross-cutting technologies” call.

## Engineering Prokaryotes

The second scientific session moved away from reconstituted systems and toward living cells and prokaryotic organisms. Michael Brockhurst (University of Manchester, UK) started by discussing the engineering of plasmid mobile elements and phage. He highlighted how “mobile elements are nature’s microbiome engineers”, driving the spread of functional traits between species, including biotechnologically useful functions such as the bioremediation of pollutants.^7^ He went on to discuss the power of bacteriophage engineering for applications in medicine and for the *in situ* control of bacterial communities, and how ecological and evolutionary thinking can be used to design better treatments.^8,9^ Brockhurst stressed how we tend to focus on what is seen as the “living” component of a biosystem, but that mobile, self-replicating molecular elements play a huge role in the regulation of many processes in microbial communities, and that horizontal gene transfer and ecological interactions are often key for microbiome stability.^10^

Following this, the session then considered how movement in prokaryotes might be engineered from the bottom-up, with Hana Kiyama (Osaka Metropolitan University, Japan) demonstrating the ability to reconstitute swimming motility in a minimal cell (JCVI-syn3B).^11^ She showed how only a few elements were needed to enable this seemly complex functionality, with two bacterial actins from Spiroplasma causing a dramatic change in the morphology of JCVI-syn3B and the generation of cellular motion. She also stressed that the ability to combine biological functions from diverse organisms offered much promise for co-opting useful phenotypes already present in nature for new applications and needs.

Continuing this theme, Makoto Miyata (Osaka Metropolitan University, Japan) then discussed the origins of mobility in life^12^ and delved further into the molecular mechanisms that allowed for motility to be reconstituted in JCVI-syn3B. He showed how the dimeric assembly of an F1-like ATPase was sufficient for gliding motility of Mycoplasma^13^ and used a broad range of structural techniques including cryo-electron microscopy (cryo-EM) and single molecule imaging to tease apart the core mechanism. The talk included some fascinating movies that demonstrated how even metabolically dead cells could have motility activated via the addition of ATP.

Talks then shifted toward industrial applications, with Masahito Ishikawa (Nagahama Institute of BioScience and Technology, Japan) introducing new approaches for engineering Acinetobacter sp. Tol 5 as a versatile chassis for biotechnology. He explained how Tol 5 was a perfect chassis to produce organic compounds due to its metabolic versatility and ability to produce strong adhesive nanofibers to immobilize cells.^14,15^ However, he also highlighted the major difficulties transforming this organism and the work his group had done to overcome this hurdle. Specially, he showed how deactivating defense mechanisms (e.g., DNA modification/restriction systems) could enable the efficient engineering of these cells using standard CRISPR-Cas editing technologies.^16^ He further suggested that this approach may be generally applicable to the domestication of other difficult to work with microorganisms, opening avenues to better harness biodiversity.

## Engineering Eukaryotes

The next session looked at eukaryotic cells and the emerging approaches for engineering these organisms and their genomes. It began with Hirohide Saito (Kyoto University and The University of Tokyo, Japan) discussing RNA synthetic biology for mammalian cell programming. He showed the power of evolutionary and generative AI approaches for designing diverse RNA families.^17^ Crucially, the RfamGen deep generative model his group developed was able to design not only functional sequences, but in some cases, sequences that outperformed their natural counterparts. With RNA synthetic biology offering many advantages over typical cellular programming techniques (e.g., those based on protein-based transcription factors), this work demonstrated the growing potential for engineering purely RNA-based systems via emerging AI tools.

Yuka Iwasaki (RIKEN, Japan) ended the first day by speaking about the role of transcriptional silencing by small noncoding RNAs. She began by highlighting the large number of types of small RNA that refine gene regulation in eukaryotes, placing a focus on piwi-interacting RNAs (piRNAs).^18,19^ She showed how in Drosophila, piRNAs can be artificially designed and expressed^20^ and went on to demonstrate the targeting rule of piRNAs and that the Drosophila Piwi-piRNA complexes distinguish transposons from mRNAs based on piRNA complementarity and abundance.^21^ She highlighted that this mechanism is particularly important when utilizing piRNAs as a novel tool for artificially inducing heterochromatin formation or for the silencing of transposable elements

The program for the second day continued with a focus on eukaryotic cells. Ben Lehner (The Wellcome Sanger Institute, UK) started proceedings by advocating for us to “mutate everything”, showing the immense power of site-saturation mutagenesis coupled to selection and sequencing. By applying this type of approach to the GTPase KRAS, which is thought to play an important role in around 10% of cancers, he showed how more that 22,000 biophysical measurements of free energy changes could be made to understand the role of mutations in tuning binding specificity and regulation at allosteric sites.^22^ Such detailed measurements are rare but offer unique insights into how protein function might be best modified and the small molecules that might facilitate such changes. He ended by touching on an even more ambitious application of the approach where over 500 human protein domains were assessed for stability and showed how these large data sets can be combined with large protein language models to enable the automatic annotation of functionally important sites.^23^

Next, Nozomu Yachie (University of British Columbia, Canada, and Osaka University, Japan) introduced a major challenge in mammalian biology. Specifically, difficulties in monitoring the cellular dynamics of multicellular organisms due to omics technologies requiring the destruction of the sample, thereby precluding time-course analyses. To overcome this, he proposed the idea of “DNA event recording” that allowed for a record of molecular and cellular events to be stored in a synthetic DNA memory sequences.^24^ He focused on several new technologies developed by his lab, including CRISPR base editing tools^25^ and a supporting deep distributed computing framework to reconstruct extremely large lineage trees.^26^ He also introduced their recent retrospective clone isolation technology as a complementary idea to DNA event recording,^27^ discussed a cell barcoding system using CRISPR base editing and showed how a target barcoded clone can be isolated from a complex population. Diverse applications of the technology for mammalian, yeast and bacterial cells were presented, including the isolation of elite human pluripotent stem cell clones that have a high propensity for naive stem cell induction.

This was followed by Yasunori Aizawa (Tokyo Institute of Technology, Japan) who introduced the idea of large-scale human genome engineering for basic science and therapy. Focusing specifically on noncoding DNA, he introduced the Universal Knock-in System (UKiS), which allows for scarless, biallelic, and gene-wide modifications.^28^ Using this system, he was able to assess the functional significance of several introns in human genes, showing their important role in boosting gene expression. He ended by discussing the commercial impacts of this technology through his spin out company Logomix, and the increasingly important role of automation in enabling the scale-up of these tools for genome engineering.

Francesca Ceroni (Imperial College London, UK) then kicked off the second part of the session by highlighting the need for more host-aware engineering approaches to synthetic biology that can help overcome the unavoidable issue of resource competition and cellular burden.^29^ Building on her original work on cell burden and its mitigation in prokaryotes,^30^ she showed how the same problem affects both transiently and stably engineered mammalian cells and suggested that construct design considerations and host-aware regulatory tools can help optimize mammalian cell engineering efforts.^31,32^

Takashi Ito (Kyusyu University, Japan) ended the session speaking about engineering gene duplications via Cas9 nickase-mediated replication fork breakage. He first showed how dCas9 can impede replication fork progression, destabilizing tandem repeats and causing copy number variation.^33^ He then went on to demonstrate how Cas9 nickase enzymes directed either side of a replication origin can cause targeted duplication of segments up to 1 Mb in size.^34^ He stressed that while CRISPR-Cas technologies have been shown capable of smaller edits and modifications, these results highlight its promise for exploring more significant structural variation.

## Fundamentals and Foundational Technologies

The final scientific session of the conference explored some of the more fundamental and foundational technologies that would be necessary to support the development of synthetic biology beyond today’s capabilities. It began with Thomas Gorochowski (University of Bristol, UK) demonstrating the need for better ways of monitoring genetic circuit function in living cells. He showed how RNA sequencing and ribosome profiling can characterize entire synthetic genetic circuits^35,36^ and the ability to use nanopore-based direct-RNA sequencing to perform massively parallel characterization of libraries of regulatory parts.^37^ Finally, he touched upon recent work that harnessed these large data sets for training machine learning models able to predict gene expression from DNA sequence and discussed ways that transfer learning can be used to alleviate the need for large and expensive experiments to retrain these models when moving between genetic, cellular, and environmental contexts.^38^

Next, Masato Kanemaki (National Institute of Genetics, Japan) began by describing an auxin-inducible degron system in mammalian cells offering precise control over gene expression.^39^ He then discussed more recent work aimed at creating an artificial replication origin in human cells. This required dissecting native DNA replication processes and covered a broad array of interesting sequencing approaches for probing this process.^40^

This was followed by Tatsuo Fukagawa (Osaka University, Japan) who spoke about the creation of an artificial kinetochore to understand their assembly mechanism and origins in human cells.^41^ He discussed the key role of the kinetochore in cell division, and demonstrated the power of using synthetic biology to more deeply understand fundamental cellular processes.^42,43^

Patrick Yizhi Cai (University of Manchester, UK) then gave a wide-ranging talk on the development of synthetic genomics in the context of the international Sc2.0 project.^44^ He showed how a secondary SCRaMbLE system had been included in the construction of the tRNA neochromosome to explore aspects of protein translation via structural rearrangements, and illustrated how diverse sequencing assays (e.g., Hi-C) had enabled his team to gain a deeper understanding of the physical positioning and function of the neochromosome in living cells.^45^ He ended by discussing some of his more recent efforts to develop effective biocontainment strategies by employing synthetic genomics and some of the key considerations when deploying synthetic biology into the real world.^46^

Next, Moe Yabuta (The University of Tokyo, Japan) spoke about self-growing protocell models. These consisted of dextran-rich droplets in an aqueous two-phase mix of polyethylene glycol and dextran. Their growth could be stimulated by internal replication of DNA via a cell-free transcription-translation system, offering yet another platform for exploring cell growth and division.^47^

The session and conference ended with an engaging talk by Joy Zhang (University of Kent, UK) that focused on what she called “care-full synthesis” and how responsible research and innovation (RRI) can help lead to better science and better policies for all involved.^48^ She highlighted the crucial need for global engagement due to the planetary impact of synthetic biology technologies and highlighted several of the current challenges when working internationally due to changes in stances on collaboration and engagement.

## Summary and Outlook

Overall, the conference offered a glimpse at the broad range of synthetic biology research being carried out across Japan and the UK. Several common themes resonated through many of the talks. These included: (i) the ability for synthetic biology to tease apart biological complexity and answer deep questions about how biology works via bottom-up reconstitution and top-down high-throughput assays with engineered cells; (ii) a need to address difficulties in scaling the complexity of the biological systems we build; and (iii) interest in the emerging role of AI methods for bridging gaps in our understanding and to capture subtle biological relationships more effectively.

Many of the talks from both countries focused on using synthetic biology to perform basic discovery science and address fundamental questions in biology. However, it was also evident that UK-based research tended to have a keener focus on translation of these results into real-world impact on shorter time scales, with significant investments in national infrastructure (e.g., biofoundries) to support this goal. There was also a clear growing interest from both countries in terms of understanding and controlling risks associated with emerging synthetic biology technologies. Providing bilateral funding to support strategic collaborations that bridge the responsible scale-up and translation of basic synthetic biology science could offer valuable and complementary points of interaction to further stimulate the synthetic biology communities in both countries and make the most of the differing perspectives they bring to the field.

Looking forward, a follow-up conference is being planning for September 2025 in the UK (Manchester). Given success of this event, it is sure to stimulate yet further connections, scientific links, and collaborations between Japanese and UK researchers and support their efforts to more deeply understand and engineer biology.

## Supplementary Material



## Notes

Table S1: List of participants, talks, and associated references (PDF)
