# Supplementary material for: The Japan-UK Synthetic Biology Conference, Spring 2025: Strengthening Global Links to Engineer Biology
Source: ACS Synth Biol. 2025 Jun 20;14(6):1873–8. doi: 10.1021/acssynbio.5c00232 (PMC12186667; doi:10.1021/acssynbio.5c00232)
Supplement: Supplementary file 1 [file sb5c00232_si_001.pdf]

Supporting Information for:

The Japan-UK Synthetic Biology Conference, Spring 2025: strengthening global links to engineer biology

Thomas E. Gorochowski[1], Michael A. Brockhurst[2], Francesca Ceroni[3,4,5], Yuka W. Iwasaki[6] and Nozomu Yachie[7,8,9]

[1] School of Biological Sciences, University of Bristol, 24 Tyndall Avenue, Bristol, BS8 1TQ, UK

[2] Division of Evolution, Infection and Genomics, Faculty of Biology, Medicine and Health, University of Manchester, Manchester, M13 9PL, UK

[3] Department of Chemical Engineering and Imperial College Centre for Synthetic Biology Imperial College London, London, SW7 2AZ, UK

[4] Imperial College Centre for Synthetic Biology, Imperial College London, SW7 2AZ, London, UK

[5] Bezos Centre for Sustainable Proteins and National Alternative Protein Innovation Centre (NAPIC), London, SW7 2AZ, UK

[6] Laboratory for Functional Non-coding Genomics, RIKEN Center for Integrative Medical Sciences, 1-7-22 Suehiro-cho, Tsurumi-ku, Yokohama 230-0045, Japan

[7] School of Biomedical Engineering, Faculty of Applied Science and Faculty of Medicine, The University of British Columbia, Vancouver, British Columbia V6T 1Z3, Canada

[8] Premium Research Institute for Human Metaverse Medicine (WPI-PRIMe), Osaka University, Suita, Osaka 565-0871, Japan

[9] Research Center for Advanced Science and Technology, The University of Tokyo, Tokyo 153-8904, Japan

Table of Contents

	Page
Table S1: List of participants, talks and associated references	2
Supplementary References	3

Table S1: List of participants, talks and associated references

Participant	Affiliation	Talk title	Refs.
**Japan**			
Hajime Niwa	Japan Science and Technology Agency, Japan	–	–
Hana Kiyama	Osaka Metropolitan University, Japan	Reconstitution of bacterial swimming motility in a minimal synthetic bacterium JCVI-syn3B	[1]
Harukiko Siomi	Keio University, Japan	–	–
Hirohide Saito	Kyoto University, Japan; University of Tokyo, Japan	RNA synthetic biology for mammalian cell programming	[2]
Makoto Miyata	Osaka Metropolitan University, Japan	Motility and life origins clarified by a minimal synthetic bacterium JCVI-syn3B	[3]
Masahito Ishikawa	Nagahama Institute of Bio Science and Technology, Japan	Cell engineering of *Acinetobacter* sp. Tol 5 as a new bacterial chassis	[4], [5]
Masato Kanemaki	National Institute of Genetics, Japan	DNA replication origins in human cells: How can we construct an artificial replication origin?	[6], [7]
Moe Yabuta	University of Tokyo, Japan	Self-growing protocell models in aqueous two-phase system induced by internal DNA replication reaction	[8]
Norikazu Ichihashi	University of Tokyo, Japan	Towards an in vitro central dogma	[9], [10]
Nozomu Yachie	University of British Columbia, Canda; Osaka University, Japan	An HD video recorder for cells	[11], [12], [13]
Takashi Ito	Kyusyu University, Japan	Engineering gene duplications via Cas9 nickase-mediated replication fork breakage	[14], [15]
Tatsuo Fukagawa	Osaka University, Japan	Artificial generation of the kinetochore to understand its assembly mechanism	[16], [17]
Yuka Iwasaki	RIKEN, Japan	Transcriptional silencing by small non-coding RNAs	[18], [19]
Yasunori Aizawa	Tokyo Institute of Technology, Japan	Large-scale human genome engineering for basic science & therapy	[20]
Yutetsu Kuruma	Japan Agency for Marine-Earth Science and Technology, Japan	Design and construction of sustainably functioning artificial cells based on cell-free system	[21]
Yuta Kawashima	Japan Science and Technology Agency, Japan	JST's programme and activities for international joint research and mobility	–
Kunio Okawa	Japan Science and Technology Agency, Japan	JST's programme and activities for international joint research and mobility	–
**United Kingdom**			
Ben Lehner	The Wellcome Sanger Institute, UK	Mutate everything	[22], [23]
Francesca Ceroni	Imperial College London, UK	Host-aware cell engineering tools	[24], [25]
Joy Zhang	University of Kent, UK	Care-full synthesis: How RRI can help lead to better science and better policies	[26]
Michael Brockhurst	University of Manchester, UK	Engineered mobile genetic elements and phages for microbiome manipulation	[27], [28], [29]
Patrick Yizhi Cai	University of Manchester, UK	Engineering tRNA neochromosomes	[30], [31]
Paul Freemont	Imperial College London, UK	Cell-free expression: a strategy for prototyping parts, pathways, xenobiotics and reconstituting complex systems for different biotechnology applications	[32], [33], [34]
Thomas Gorochowski	University of Bristol, UK	Sequencing-based methods for accelerated cellular programming	[35], [36], [37]

Supplementary References

[1] H. Kiyama, S. Kakizawa, Y. Sasajima, Y. O. Tahara, and M. Miyata, 'Reconstitution of a minimal motility system based on *Spiroplasma* swimming by two bacterial actins in a synthetic minimal bacterium', *Sci. Adv.*, vol. 8, no. 48, p. eabo7490, Dec. 2022, doi: 10.1126/sciadv.abo7490.

[2] S. Sumi, M. Hamada, and H. Saito, 'Deep generative design of RNA family sequences', *Nat. Methods*, vol. 21, no. 3, pp. 435–443, Mar. 2024, doi: 10.1038/s41592-023-02148-8.

[3] T. Toyonaga *et al.*, 'Dimeric assembly of F_1 -like ATPase for the gliding motility of *Mycoplasma*', Jun. 11, 2024. doi: 10.1101/2024.06.11.597861.

[4] M. Ishikawa, T. Kojima, and K. Hori, 'Development of a Biocontained Toluene-Degrading Bacterium for Environmental Protection', *Microbiol. Spectr.*, vol. 9, no. 1, pp. e00259-21, Sep. 2021, doi: 10.1128/Spectrum.00259-21.

[5] M. Ishikawa and K. Hori, 'The elimination of two restriction enzyme genes allows for electroporation-based transformation and CRISPR-Cas9-based base editing in the non-competent Gram-negative bacterium *Acinetobacter* sp. Tol 5', *Appl. Environ. Microbiol.*, vol. 90, no. 6, pp. e00400-24, Jun. 2024, doi: 10.1128/aem.00400-24.

[6] K. Nishimura, T. Fukagawa, H. Takisawa, T. Kakimoto, and M. Kanemaki, 'An auxin-based degron system for the rapid depletion of proteins in nonplant cells', *Nat. Methods*, vol. 6, no. 12, pp. 917–922, Dec. 2009, doi: 10.1038/nmeth.1401.

[7] E. Koyanagi *et al.*, 'Global landscape of replicative DNA polymerase usage in the human genome', *Nat. Commun.*, vol. 13, no. 1, p. 7221, Nov. 2022, doi: 10.1038/s41467-022-34929-8.

[8] Y. Minagawa, M. Yabuta, M. Su'etsugu, and H. Noji, 'Self-growing protocell models in aqueous two-phase system induced by internal DNA replication reaction', *Nat. Commun.*, vol. 16, no. 1, p. 1522, Feb. 2025, doi: 10.1038/s41467-025-56172-7.

[9] K. Hagino, K. Masuda, Y. Shimizu, and N. Ichihashi, 'Sustainable Regeneration of 20 Aminoacyl-tRNA Synthetases in a Reconstituted System Toward Self-Synthesizing Artificial Systems', Oct. 03, 2024. doi: 10.1101/2024.10.03.616507.

[10] R. Miyachi, Y. Shimizu, and N. Ichihashi, 'Simultaneous in vitro expression of minimal 21 transfer RNAs by tRNA array method', Feb. 15, 2025. doi: 10.1101/2025.02.15.638384.

[11] K. Nishida *et al.*, 'Targeted nucleotide editing using hybrid prokaryotic and vertebrate adaptive immune systems', *Science*, vol. 353, no. 6305, p. aaf8729, Sep. 2016, doi: 10.1126/science.aaf8729.

[12] N. Konno *et al.*, 'Deep distributed computing to reconstruct extremely large lineage trees', *Nat. Biotechnol.*, vol. 40, no. 4, pp. 566–575, Apr. 2022, doi: 10.1038/s41587-021-01111-2.

[13] S. Ishiguro *et al.*, 'A multi-kingdom genetic barcoding system for precise target clone isolation', Jan. 19, 2023. doi: 10.1101/2023.01.18.524633.

[14] G. Doi *et al.*, ‘Catalytically inactive Cas9 impairs DNA replication fork progression to induce focal genomic instability’, *Nucleic Acids Res.*, vol. 49, no. 2, pp. 954–968, Jan. 2021, doi: 10.1093/nar/gkaa1241.

[15] Y. Sugiyama, S. Okada, Y. Daigaku, E. Kusumoto, and T. Ito, ‘Strategic targeting of Cas9 nickase induces large segmental duplications’, *Cell Genomics*, vol. 4, no. 8, p. 100610, Aug. 2024, doi: 10.1016/j.xgen.2024.100610.

[16] T. Hori and T. Fukagawa, ‘Artificial generation of centromeres and kinetochores to understand their structure and function’, *Exp. Cell Res.*, vol. 389, no. 2, p. 111898, Apr. 2020, doi: 10.1016/j.yexcr.2020.111898.

[17] S. Sridhar and T. Fukagawa, ‘Meiosis: When centromeres choose compromise over conflict’, *Curr. Biol.*, vol. 35, no. 5, pp. R196–R198, Mar. 2025, doi: 10.1016/j.cub.2025.01.059.

[18] H. Ishizu, T. Kinoshita, S. Hirakata, C. Komatsuzaki, and M. C. Siomi, ‘Distinct and Collaborative Functions of Yb and Armitage in Transposon-Targeting piRNA Biogenesis’, *Cell Rep.*, vol. 27, no. 6, pp. 1822-1835.e8, May 2019, doi: 10.1016/j.celrep.2019.04.029.

[19] M. Ariura *et al.*, ‘Drosophila Piwi distinguishes transposons from mRNAs by piRNA complementarity and abundance’, *Cell Rep.*, vol. 43, no. 12, p. 115020, Dec. 2024, doi: 10.1016/j.celrep.2024.115020.

[20] T. Ohno *et al.*, ‘Biallelic and gene-wide genomic substitution for endogenous intron and retroelement mutagenesis in human cells’, *Nat. Commun.*, vol. 13, no. 1, p. 4219, Jul. 2022, doi: 10.1038/s41467-022-31982-1.

[21] S. Eto *et al.*, ‘Phospholipid synthesis inside phospholipid membrane vesicles’, *Commun. Biol.*, vol. 5, no. 1, p. 1016, Sep. 2022, doi: 10.1038/s42003-022-03999-1.

[22] C. Weng, A. J. Faure, A. Escobedo, and B. Lehner, ‘The energetic and allosteric landscape for KRAS inhibition’, *Nature*, vol. 626, no. 7999, pp. 643–652, Feb. 2024, doi: 10.1038/s41586-023-06954-0.

[23] A. Beltran, X. Jiang, Y. Shen, and B. Lehner, ‘Site-saturation mutagenesis of 500 human protein domains’, *Nature*, vol. 637, no. 8047, pp. 885–894, Jan. 2025, doi: 10.1038/s41586-024-08370-4.

[24] J. Gabrielli, R. Di Blasi, C. Kontoravdi, and F. Ceroni, ‘Degradation bottlenecks and resource competition in transiently and stably engineered mammalian cells’, *Nat. Commun.*, vol. 16, no. 1, p. 328, Jan. 2025, doi: 10.1038/s41467-024-55311-w.

[25] A. Zouein, B. Lende-Dorn, K. E. Galloway, T. Ellis, and F. Ceroni, ‘Engineered Transcription Factor Binding Arrays for DNA-based Gene Expression Control in Mammalian Cells’, Sep. 03, 2024. doi: 10.1101/2024.09.03.610999.

[26] S. K. Namdeo and J. Y. Zhang, ‘Rethinking science diplomacy and global biosecurity: challenges, emerging practices and the way forward’, *Int. Aff.*, vol. 100, no. 6, pp. 2623–2635, Nov. 2024, doi: 10.1093/ia/iiae187.

[27] R. C. T. Wright, V.-P. Friman, M. C. M. Smith, and M. A. Brockhurst, ‘Functional diversity increases the efficacy of phage combinations’, *Microbiology*, vol. 167, no. 12, Dec. 2021, doi: 10.1099/mic.0.001110.

[28] R. C. T. Wright, V.-P. Friman, M. C. M. Smith, and M. A. Brockhurst, 'Resistance Evolution against Phage Combinations Depends on the Timing and Order of Exposure', *mBio*, vol. 10, no. 5, pp. e01652-19, Oct. 2019, doi: 10.1128/mBio.01652-19.

[29] K. Z. Coyte, C. Stevenson, C. G. Knight, E. Harrison, J. P. J. Hall, and M. A. Brockhurst, 'Horizontal gene transfer and ecological interactions jointly control microbiome stability', *PLOS Biol.*, vol. 20, no. 11, p. e3001847, Nov. 2022, doi: 10.1371/journal.pbio.3001847.

[30] D. Schindler *et al.*, 'Design, construction, and functional characterization of a tRNA neochromosome in yeast', *Cell*, vol. 186, no. 24, pp. 5237-5253.e22, Nov. 2023, doi: 10.1016/j.cell.2023.10.015.

[31] S. A. Hoffmann and Y. Cai, 'Engineering stringent genetic biocontainment of yeast with a protein stability switch', *Nat. Commun.*, vol. 15, no. 1, p. 1060, Feb. 2024, doi: 10.1038/s41467-024-44988-8.

[32] Y. Zhang *et al.*, 'Optimizing protein production in the One-Pot Pure system: insights into reaction composition and expression efficiency', Jun. 19, 2024. doi: 10.1101/2024.06.19.599772.

[33] R. J. R. Kelwick, A. J. Webb, and P. S. Freemont, 'Accelerating extracellular vesicle research and biotechnological applications using synthetic biology approaches', *Extracell. Vesicle*, vol. 4, p. 100050, Dec. 2024, doi: 10.1016/j.vesic.2024.100050.

[34] A. D. Silverman, A. S. Karim, and M. C. Jewett, 'Cell-free gene expression: an expanded repertoire of applications', *Nat. Rev. Genet.*, vol. 21, no. 3, pp. 151–170, Mar. 2020, doi: 10.1038/s41576-019-0186-3.

[35] T. E. Gorochowski *et al.*, 'Genetic circuit characterization and debugging using RNA-seq', *Mol. Syst. Biol.*, vol. 13, no. 11, p. 952, Nov. 2017, doi: 10.15252/msb.20167461.

[36] T. E. Gorochowski, I. Chelysheva, M. Eriksen, P. Nair, S. Pedersen, and Z. Ignatova, 'Absolute quantification of translational regulation and burden using combined sequencing approaches', *Mol. Syst. Biol.*, vol. 15, no. 5, p. e8719, May 2019, doi: 10.15252/msb.20188719.

[37] M. J. Tarnowski and T. E. Gorochowski, 'Massively parallel characterization of engineered transcript isoforms using direct RNA sequencing', *Nat. Commun.*, vol. 13, no. 1, p. 434, Jan. 2022, doi: 10.1038/s41467-022-28074-5.
